# Mutational load analysis of unrelated individuals

**DOI:** 10.1186/1753-6561-5-S9-S55

**Published:** 2011-11-29

**Authors:** Daniel P Howrigan, Matthew A Simonson, Helen M Kamens, Sarah H Stephens, Amanda G Wills, Marissa A Ehringer, Matthew C Keller, Matthew B McQueen

**Affiliations:** 1 Institute for Behavioral Genetics, University of Colorado at Boulder, 1480 30th Street, Boulder, CO 80303, USA; 2 Department of Psychology, University of Colorado at Boulder, 345 UCB, Boulder, CO 80309-0345, USA; 3 Department of Integrative Physiology, University of Colorado at Boulder, Clare Small 113, 354 UCB, Boulder, CO 80309-0354, USA

## Abstract

Evolutionary genetic models predict that the cumulative effect of rare deleterious mutations across the genome—known as mutational load burden—increases the susceptibility to complex disease. To test the mutational load burden hypothesis, we adopted a two-tiered approach: assessing the impact of whole-exome minor allele load burden and then conducting individual-gene screening. For our primary analysis, we examined various minor allele frequency (MAF) thresholds and weighting schemes to examine the overall effect of minor allele load on affection status. We found a consistent association between minor allele load and affection status, but this effect did not markedly increase within rare and/or functional single-nucleotide polymorphisms (SNPs). Our follow-up analysis considered minor allele load in individual genes to see whether only one or a few genes were driving the overall effect. Examining our most significant result—minor allele load of nonsynonymous SNPs with MAF < 2.4%—we detected no significantly associated genes after Bonferroni correction for multiple testing. After moderately significant genes (*p* < 0.05) were removed, the overall effect of rare nonsynonymous allele load remained significant. Overall, we did not find clear support for mutational load burden on affection status; however, these results are ultimately dependent on and limited by the nature of the Genetic Analysis Workshop 17 simulation.

## Background

The advent of next-generation sequencing technology has enabled researchers to detect rare genetic variation. Despite this technological advance, detecting low-frequency susceptibility alleles that underlie common disorders has inherent difficulties. In general, detection of a true signal is statistically challenging when any given variant is observed in only one or a few individuals. Furthermore, many variants of large effect and/or complete penetrance, such as those underlying Mendelian disorders, are more likely to be identified using traditional linkage methods. This poses a serious challenge to the discovery of any particular causal variant, because rare variants of small effect and/or incomplete penetrance will be difficult to detect using traditional association methods.

An alternative approach is to test overall mutational load across individuals, because variation observed in complex disorders may be partly explained by the cumulative effects of rare deleterious mutations scattered across a large number of genes [1-4]. Although any single mutation might be present in only one or a few individuals in a given sample (e.g., a rare allele, a single-nucleotide polymorphism [SNP] with low minor allele frequency [MAF]) and likely has a negligible effect (if any) on the overall trait variation, the overall load of mutations is much more likely to disrupt traits that rely on the proper functioning of many genes. Support for this approach comes from evolutionary genetic models of mutation-selection balance, where the equilibrium between the introduction of deleterious mutations in the population and their eventual removal by natural selection may take hundreds of generations [5-7]. For the purposes of this study, our mutational load model makes two specific predictions for analyzing sequence data: (1) Trait disorder will associate most strongly when the total load of rare variants is assessed, and (2) this association will not be explained by variants in just one or a few genes.

To examine mutational load, we assessed the cumulative effect of minor alleles associated with affection status among unrelated individuals in the Genetic Analysis Workshop 17 (GAW17) mini-exome data set. In contrast to traditional association analyses that test the effect of an individual allele on a phenotype, our approach collapses across the overall load (or sum) of minor alleles carried by an individual. Such analyses are also referred to as pooled association tests [8], which is a subtype of broader collapsing methods [9]. Our hypothesis is that individuals with a higher load of minor alleles will be associated with disorder-related phenotypes, and alleles with a lower MAF and increased functional effects will largely contribute to this association. Because there is no specific MAF threshold that distinguishes mutations from the full set of polymorphisms, we use two thresholds (MAF < 0.05 and MAF < 0.01) to assess whether increasingly rare SNPs show a stronger association with affection status. Our analysis uses various techniques from Price et al. [8] to conduct minor allele load tests on the entire exome data set. This is followed by a minor allele load analysis within individual genes to check whether detected overall effects, particularly those of rare functional SNPs, can be explained by a single gene or a small subset of genes in the sample.

## Methods

### Data

All analyses were carried out using R statistical software, version 2.11.1 (http://www.r-project.org), and PLINK, version 1.07 [10]. To simplify the approach, we examined only data from unrelated subjects (*n* = 697; 327 males and 370 females). We reformatted the genotype and phenotype data to assemble the input files required by PLINK. Because the data were imputed for SNP missingness and because most SNPs occur at low frequency, we did not conduct any data cleaning procedures.

We focused on affection status as the primary phenotype of interest. To avoid any bias that may arise from any single replicate of the phenotypes, we aggregated affection status for each individual across all 200 phenotype replicates. We then designated the top 30% of aggregated scores as affected (just as the top 30% of latent liability scores are designated as affected in each phenotype replicate) to return the phenotype to a case-control paradigm. Because the three quantitative phenotypes were used to construct the affection status scores, we did not take into account their unique effects in this study.

**Figure 1:**
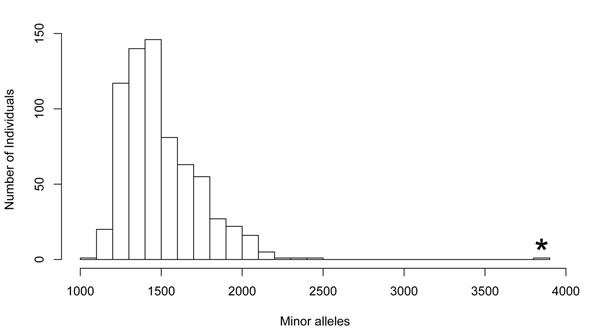
**Distribution of minor allele load** One subject, NA19237, was found to have a minor allele load about 10 standard deviations above the mean minor allele load. The asterisk indicates this outlier. After the outlier was removed, the mean allele count was 1,496 (SD = 224).

Before data analysis, we examined the distribution of overall minor allele load for possible outliers. We used the --profile command in PLINK to compute the minor allele load. This command provides a count of minor alleles per individual along with a scoring procedure to give various weights to certain SNPs. We also used the --profile command in subsequent pooled association analyses. Figure 1 shows the distribution of minor allele load in the data. There is one clear outlier, NA19237, which was about 10 standard deviations above the mean minor allele load. We removed the data for NA19237 before our analysis.

We were also interested in the relationship of affection status to the fixed covariates of Age, Sex, and Ethnicity (smoking status was excluded because it varied across phenotype replicates). Table 1 shows the correlation matrix of affection status with the three covariates. The strong relationship between Age and affection status raised concerns about colinearity with affection status, so Age was excluded as a covariate in our analysis.

**Table 1:** Correlation matrix of affection status and fixed covariates

	Affected	Sex	Age
Sex	0.03		
Age	0.75*	0.01	
Ethnicity	0.17*	0.00	0.20*

* *p* < 0.001.

### Whole-exome minor allele load analysis

The whole-exome analyses consisted of both threshold and weighting procedures to test the association of minor allele load to affection status across the entire exome. Table 2 lists the various procedures used, many of which are adopted from Price et al. [8].

**Table 2:** Minor allele load procedures

Procedure	Analytic description
All SNPs	All minor alleles counted
MAF ≥ 0.05	Only alleles with MAF ≥ 0.05 counted (T)
MAF < 0.05	Only alleles with MAF < 0.05 counted (T)
MAF < 0.01	Only alleles with MAF < 0.01 counted (T)
Inverse weighting	Exponentially increasing weight to rarer alleles (W)
Variable threshold	Empirically defined MAF level used as threshold. Significance drawn through permutation analysis (T)
Nonsynonymous/synonymous	Nonsynonymous and synonymous SNPs examined separately (F)
Functional weighting	SNPs weighted by degree of amino acid change derived from BLOSUM (WF)

T, threshold procedure; W, weighting procedure; F, function-based procedure.

Each of the procedures used the PLINK --profile command to calculate allele counts or scores for each individual. The inverse weighting scheme used the weighting algorithm 1/[*q*(1 − *q*)]^1/2^ for each variant, where *q* is the allele frequency of the minor allele in the sample. For the variable threshold procedure, we examined all possible MAF thresholds to find the most significant threshold. To verify the significance of this procedure, we permuted affection status 1,000 times, selecting the most significant MAF threshold *p*-value from each permutation to build a distribution of empirical *p*-values. To incorporate functional classifications, we partitioned the data into nonsynonymous and synonymous SNP sets. In addition, we included a functional weighting algorithm that used the block substitution matrix (BLOSUM), giving a predefined score for each possible amino acid change based on the magnitude of protein divergence expected from the change [11]. Using the reference human genome build 36, we developed a program in C++ to implement the BLOSUM for the entire exome. For each functional analysis, we also incorporated a variable threshold procedure for a combined MAF threshold/functional classification minor allele load test.

We used logistic regression for each testing procedure, regressing affection status against the minor allele count or score along with Sex and Ethnicity as covariates. Ethnicity was collapsed from 17 different categories to 7 based on the larger population affiliations of each ethnic group.

### Individual-gene analysis

As a follow-up to the whole-exome load analysis, we considered each gene individually using the most significant procedure from the whole-exome analysis to examine whether any observed overall effect was being driven primarily by a single gene or small subset of genes. The individual-gene analysis used the same logistic regression model as the whole-exome analysis, with an additional check on the accuracy of our top gene hits matching simulated causal genes. To check whether these genes were explaining the overall effect, we repeated the whole-exome test, removing significant genes, and thus determined whether the earlier effect persisted in the remaining exome.

## Results

### Whole-exome minor allele load

Table 3 shows the results from the logistic regression analysis on affection status using the whole-exome SNP set. Table 4 shows the results using the variable threshold method, where the most significant MAF threshold was used and validated with empirical *p*-values from a permutation distribution of affection status. In each table, the odds ratio for minor allele load is the standardized odds of being affected for each standard deviation from the mean allele count or score in the sample. The strong effect of ethnicity in the data was unexpected but likely stems from differences in allele frequencies of causal SNPs in some ethnic groups, because there is no mention of simulated ethnicity effects in the GAW17 answers.

**Table 3:** Whole-exome minor allele load results

Procedure	Number of SNPs	Minor allele load		Sex		Ethnicity	
		
		Odds ratio	*p*-value	Odds ratio	*p*-value	Odds ratio	*p*-value
All SNPs	24,487	1.296	2.05 × 10^−3^	1.096	0.28	1.405	7.15 × 10^−5^
MAF ≥ 0.05	3,126	1.247	8.63 × 10^−3^	1.093	0.30	1.514	1.59 × 10^−5^
MAF < 0.05	21,361	1.338	1.25 × 10^−3^	1.088	0.32	1.289	6.12 × 10^−3^
MAF < 0.01	18,175	1.326	2.68 × 10^−3^	1.093	0.30	1.274	0.018
Inverse weighting	24,287	1.329	1.22 × 10^−3^	1.095	0.29	1.323	1.71 × 10^−3^
Nonsynonymous	13,572	1.287	2.36 × 10^−3^	1.090	0.31	1.426	3.17 × 10^−5^
Synonymous	10,113	1.311	1.81 × 10^−3^	1.099	0.27	1.360	4.06 × 10^−4^
BLOSUM weighting	24,487	1.296	2.00 × 10^−3^	1.094	0.29	1.404	7.38 × 10^−5^

Each row represents a different minor allele load procedure. The number of SNPs analyzed depends on the inclusion threshold or functional category for each procedure.

**Table 4:** Whole-exome variable threshold results

			Minor allele load		
			
Procedure	MAF threshold	Number of SNPs	Odds ratio	Observed *p*-value	Empirical *p*-value
All SNPs	0.0065	17,207	1.350	7.51 × 10^−4^	0.002
Nonsynonymous	0.023	11,647	1.401	3.45 × 10^−4^	0.001
Synonymous	0.405	9,951	1.321	1.49 × 10^−3^	0.004
BLOSUM weighting	0.144	22,759	1.355	8.10 × 10^−4^	0.002

The variable threshold procedure examines all MAF thresholds to find the most significant threshold. Empirical *p*-values are derived from 1,000 permutations of affection status.

In general, the various thresholds and weighting procedures show a consistent main effect of minor allele load associated with affection status. The direction of effect is also consistent across procedures, with the probability of affection (control = 0, case = 1) slightly increasing with higher load and/or score. Although no procedure shows a markedly stronger effect above the others, the most significant effect is observed for the variable threshold procedure using nonsynonymous SNPs with MAF < 0.024 (OR = 1.401, observed *p* = 7.1 × 10^−4^, empirical *p* = 0.001). BLOSUM functional scoring shows a similar effect to nonsynonymous SNPs, which is not surprising because the GAW17 answers do not incorporate the magnitude of nonsynonymous amino acid changes into their effect sizes. Considering only MAF threshold, we find that the variable threshold effect is maximized at a MAF below 5%, but lowering the MAF threshold does not consistently increase the effect of minor allele load, as noted by the slightly lower *p*-value of the MAF < 0.05 threshold (*p* = 0.001) compared to the MAF < 0.01 threshold (*p* = 0.002). Contrary to the predictions of a mutational load model, we do not see a marked increase in the strength of association when looking specifically at rare SNPs (MAF < 0.01), functional SNPs, or a combination of both; we find only a marginal increase in association strength with low-MAF nonsynonymous SNPs using the variable threshold procedure.

All the simulated causal SNPs in the GAW17 answers are nonsynonymous SNPs, so it is interesting to observe a slightly stronger effect in overall synonymous minor allele load (*p* = 1.81 × 10^−3^) relative to overall nonsynonymous minor allele load (*p* = 2.36 × 10^−3^). Furthermore, the large discrepancy in optimal MAF when using the variable threshold procedure (optimal synonymous MAF = 0.404; optimal nonsynonymous MAF = 0.023) suggests that different mechanisms underlie the observed effects. Given that power to detect an effect increases with higher MAF and that linkage disequilibrium creates associations with nearby noncausal SNPs, it is possible that synonymous SNPs with higher MAF are in linkage disequilibrium with causal SNPs and are driving the observed effect. A comparison of nonsynonymous SNP allele frequencies (mean MAF = 0.025) with synonymous SNP allele frequencies (mean MAF = 0.037) shows a significant difference: *t*(23,683) = 11.24, *p* < 2 × 10^−16^. Although this difference suggests that a higher average MAF may be causing the synonymous minor allele load effect, fine-scale haplotype and single-SNP linkage disequilibrium patterns provide more conclusive evidence that high-MAF synonymous SNPs in linkage disequilibrium with causal SNPs are driving the observed signal.

### Individual-gene minor allele load

Given that significant association is found between overall minor allele load and affection status, we wanted to test whether this result was driven by the cumulative effect of minor alleles or whether it was the result of a few genes. Using the strongest result, the variable threshold procedure within the nonsynonymous SNP set, we analyzed minor allele load within each individual gene. To maintain the consistency of each test, we did not look for the optimal MAF threshold for each gene but used the overall MAF < 0.024 threshold for all genes. We found that 1,009 genes did not contain any nonsynonymous SNPs and that an additional 341 genes did not contain any SNPs with MAF < 0.024, so we limited the analysis to the remaining 1,855 genes. Using the Bonferroni-corrected *p*-value for multiple testing, we set the genome-wide significance threshold at 0.05/1,855 = 2.7 × 10^−5^.

We did not find any genes that surpassed Bonferroni correction; the top gene, *PIK3C2B* (OR = 1.33, *p* = 3.5 × 10^−4^), reached significance at an order of magnitude below Bonferroni correction. Although this gene and the second top hit, *FLT1* (OR = 1.31, *p* = 6.6 × 10^−4^), were both simulated as causal genes, only 4 of the 36 simulated causal genes were detected among the 96 genes with *p* < 0.05. This corresponds to a false-positive rate of 96% and a missing rate of 89%, showing that the most promising minor allele load procedure is highly underpowered to detect effects at the individual-gene level.

The lack of individual-gene effects also suggests that the whole-exome signal is not being driven by the action of a few genes. To confirm this, we reanalyzed the variable threshold procedure within the nonsynonymous SNP set after removing the 96 genes with *p* < 0.05. After removal, there was a markedly reduced but still significant effect in association with affection status (OR = 1.26, *p* = 0.01). However, given that most simulated causal genes were not identified in our individual-gene analysis, this result was not surprising.

## Discussion

We find that minor allele load is significantly associated with affection status across the entire exome; however, our results do not clearly support a mutational load model because the strength of association does not markedly increase when we look at only rare functional SNPs. Regardless, the strongest effect is found using nonsynonymous SNPs with MAF < 0.024, which is consistent with a model predicting that the cumulative effect of low-MAF functional SNPs is a contributing factor to the genetic variance underlying affection status. Applying this model to individual genes shows that no single gene or small subset of genes explains the whole-exome results. However, a comparison of individual-gene results with the GAW17 answers shows that minor allele load analysis of individual genes performs poorly for detecting the simulated causal genes.

## Conclusions

We designed our study with the expectation of a polygenic phenotype with knowledge of the nature of the phenotype simulation. We selected affection status as our phenotype of interest because it was influenced by all the simulated causal SNPs. In addition, we used information from all 200 phenotype replicates to form an affection status phenotype that was well suited to reflect the simulated effects of the entire suite of simulated causal SNPs. Given this approach, the significance and power of each procedure are both biased to our designed phenotype and relative to the nature of the simulation. As a consequence, the focus of this study was to assess the method for testing a mutational load model on genetic sequence data rather than significance of the association results.

## Competing interests

The authors declare that there are no competing interests.

## Authors’ contributions

DH designed the study, performed the statistical analysis, and drafted the manuscript. MS developed software to incorporate the BLOSUM matrix and helped to draft the manuscript. HK participated in the design the BLOSUM matrix and helped to draft the manuscript. SS participated in the design the BLOSUM matrix and helped to draft the manuscript. AW helped to draft the manuscript. ME participated in the design and coordination of the study and helped to draft the manuscript. MK conceived of the study and participated in its design and coordination and helped to draft the manuscript. MM conceived of the study and participated in its design and coordination and helped to draft the manuscript. All authors read and approved the final manuscript.
